# The ameliorative effect of selenium-loaded chitosan nanoparticles against silver nanoparticles-induced ovarian toxicity in female albino rats

**DOI:** 10.1186/s13048-024-01577-z

**Published:** 2025-01-07

**Authors:** Omnia E. Shalaby, Yasmine H. Ahmed, Aya M. Mekkawy, Mohamed Y. Mahmoud, G. A. Elbargeesy

**Affiliations:** 1 Department of Cytology and Histology, Faculty of Veterinary Medicine, Cairo University, Giza, Egypt; 2 Department of Toxicology and Forensic Medicine, Faculty of Veterinary Medicine, Cairo University, Giza, Egypt

**Keywords:** Ag-NPs, CS-SeNPs, Ovarian toxicit y, Histopathology, Immunohistochemistry

## Abstract

**Background:**

Recently, silver nanoparticles (Ag-NPs) were shown to provoke oxidative stress through the release of reactive oxygen species and consequently induce cell damage. Selenium-loaded chitosan nanoparticles (CS-SeNPs) have anti-inflammatory and antioxidant effects, indicating that they ameliorate Ag-NPs-induced ovarian toxicity.

**Objective:**

This study aimed to assess how well CS-SeNPs counteract the damaging effects of Ag-NPs on the ovarian tissue of adult female albino rats.

**Methods:**

Forty mature female albino rats were divided into four equal groups: for 60 days, Group I (control) was given 0.5 ml/kg of distilled water; Group II was given Ag-NPs orally (100 mg/kg); Group III was given Ag-NPs orally (100 mg/kg/d) plus CS-SeNPs (0.5 mg/kg/d); and Group IV was given only CS-SeNPs orally (0.5 mg/kg/d). All the ovarian tissues were removed and underwent immunohistochemical, histological, and biochemical analyses.

**Results:**

Ag-NPs-exposed rats revealed a marked reduction in reduced glutathione (GSH) and superoxide dismutase (SOD). Numerous histopathological alterations were found along with a significant increase in PCNA- and Caspase-3-immunoreactive cells. Most of these alterations were successfully ameliorated by CS-SeNPs, as indicated by marked increases in GSH and SOD.

**Conclusion:**

CS-SeNPs ameliorate the toxic effects of Ag-NPs on the ovarian tissue of adult female albino rats.

## Introduction

Currently, nanotechnology has attracted gravely growing interest in a wide range of disciplines, such as its use in nutrition and medicine [1, 2]. Despite the rapid development and early acceptance of nanotechnology, it is still unknown whether sustained exposure to different concentrations of nanoparticles in humans, animals, or the environment will have any negative consequences on health. In particular, the activity of nanoparticles inside cells has not been fully elucidated, and neither the metabolic nor the immunological reactions triggered by these particles are currently understood.

Recently, the unique physical, chemical, and biological features of silver nanoparticles (Ag-NPs) have increased in popularity [3–5] and their powerful antibacterial properties [6]. Ag-NPs are manipulated in almost all fields, such as medicine [7], drug delivery [8, 9], medical device coats [10], wound bandages [11], and contraceptives [12, 13]. Despite the widespread use of Ag-NPs, few reports on their toxicity exist. Several recent studies have shown that Ag-NPs provoke damage to the ovary [14]. Another study showed that Ag-NPs stop ovulation induction [15].

Selenium (Se) has potent anti-inflammatory and antioxidant activities since it is a crucial component of thirty selenoproteins, including glutathione peroxidase (GPx), three thioredoxin reductases, and selenoprotein-P [16]. Se protects cells from reactive oxygen species (ROS) insults; consequently, it prevents oxidative stress injury caused by cellular macromolecules such as membrane DNA and lipids [17]. Recently, nanosized selenium has gained increased amounts of attention because of its better bioavailability, better biocompatibility, lower toxicity, and higher surface activity than its other forms [18, 19].

Chitosan (CS) has received increased interest for its use in industrial applications in the medicine, food, and chemical sectors [20] owing to its biological functions, such as antioxidant, antiaging, and antimicrobial activities [21]. Moreover, the nano form of chitosan (CS-NPs) is increasingly used as a drug carrier due to its exclusive properties, such as slow and controlled drug release, which enhances drug stability, increases effectiveness, and decreases toxicity [22, 23]. Furthermore, coating selenium with chitosan nanoparticles prevents cellular DNA damage [24].

Ag-NPs, as previously indicated, have a wide range of uses, particularly as contraceptives with potential toxicity that may persist in the long run and based on the powerful antioxidant and anti-inflammatory effects of selenium nanoparticles (Se-NPs), which are augmented by the antioxidant and immune-stimulating abilities of chitosan (CS), It inspired us to investigate the protective role of CS-SeNPs against ovarian toxicity caused by Ag-NPs; to our knowledge, there are no published studies on the ameliorative impact of CS-SeNPs on ovarian histopathological alterations.

## Materials and methods

### Animal

From the Animal Health Research Centre in Dokki, Egypt, 40 adult female albino rats averaging 170±20 grams were obtained. The rats were kept in plastic enclosures with wood shavings and acclimated for 7 days to the laboratory environment (with a regulated humidity of 50±10% and temperature of 25±5°C) in the Department of Toxicology and Forensic Medicine at the Faculty of Veterinary Medicine, Cairo University. Water was always given to the rats, and they were nourished with a standard laboratory rat diet. The Faculty of Veterinary Medicine's institutional animal care and use committee at Cairo University approved the experimental protocol, which was conducted following its requirements (Vet. Cu. IACUC, No. 8-03-2022-435).

### Ag-NPs and CS-SeNPs synthesis and characterization: NP morphology and size

Chemical reduction was the method used for manufacturing Ag-NPs [25, 26]. The ionotropic gelation technique was used for the synthesis of selenium-loaded chitosan nanoparticles (CS-SeNPs) [27, 28]. For nanoparticle characterization, scanning electron microscopy was used to evaluate the nanoparticle diameter (XL-30 ESEMFEG SEM, FEI Company, USA). Zeta potential analysis was performed to identify the surface charge of the hydrated NPs. The encapsulation efficacy was assessed as previously described [29–31].

### Experimental design

Following the adaptation phase, forty rats were divided into the following four groups: As the control group, Group I was given a standard diet and 0.5 mL/kg distilled water. Ag-NPs (100 mg/kg) were given to Group II (Ag-NPs) [32]. Ag-NPs (100 mg/kg) and CS-SeNPs (0.5 mg/kg) were given to Group III (Ag-NPs + CS-SeNPs) [33]. The CS-SeNPs (0.5 mg/kg) were given to Group IV (CS-SeNPs). The body weights of the rats were recorded and measured every week.

## Collection of samples

Upon termination of the trial, the rats were sedated with 10 mg/kg xylazine and 60 mg/kg ketamine. Next, the rats were sacrificed by cervical dislocation. Afterward, each rat's ovaries were carefully removed, and some ovarian tissue samples were kept at −20 °C in plastic bags for biochemical investigations. The remaining specimens were kept for histopathological and immunohistochemical analyses in a 10% neutral buffered formalin solution.

## Biochemical investigation (measurement of oxidative stress parameters)

### Measurement of reduced glutathione (GSH) levels

The GSH Assay Kit (BioVision®, USA) was used to measure the GSH concentration in the ovarian tissue homogenate following the manufacturer's instructions. In brief, the procedure is based on an enzymatic cycling approach using GSH and a chromophore. The chromophore reduction creates a stable product that may be tracked kinetically at 450 nm.

### Measurement of superoxide dismutase (SOD) activity

Superoxide dismutase is one of the most significant antioxidant enzymes. It catalyzes the conversion of superoxide anions (O2 −) into molecular oxygen (O2) and hydrogen peroxide (H2O2). SOD activity in the ovarian tissue was measured by using a SOD activity assay kit (BioVision®, USA) according to the manufacturer's instructions. Briefly, the superoxide anion produced by xanthine/xanthine oxidase (XO) reduces water-soluble tetrazolium (WST-1) to formazans, which are detectable at 450 nm. The activity of xanthine oxidase (XO) determines the rate of reduction of a superoxide anion, which is suppressed by SOD. Consequently, it is possible to measure the inhibitory effect of SOD.

## Histological and histochemical examinations

### Light microscopy (LM)

All group specimens were carefully dissected, kept in 10% neutral buffered formalin for 48 h, dehydrated in ascending levels of alcohol, cleared in xylene, and finally embedded in paraffin wax. The specimens were sectioned by a rotatory microtome (at a thickness of 3 − 4 μm). After deparaffinization, the ovarian tissue samples were stained with hematoxylin and eosin (H&E) as a general routine stain and Masson’s trichrome stain for demonstrating collagen fibers [34].

### Image analysis for evaluation of histochemical observations

The blue intensity (collagen fibers) of Masson’s trichrome stain was analyzed within ten ovarian sections by using ImageJ software. The image analyzer converts the pixels into area percentages. The mean ± SD was used to express the results. A difference between groups that was statistically significant was considered to exist at *p* < 0.05.

## Immunohistochemical examination

### Caspase 3: for evaluation of apoptosis

#### Proliferating cell nuclear antigen (PCNA): for detection of mitosis

Using the avidin–biotin-peroxidase complex approach, immunohistochemical sections of ovarian tissues were obtained to identify caspase-3 and PCNA immune expression [35]. The grades for the immune response were as follows: negative (-), mild ( +), moderate (+ +), and strong (+ + +) [36]. For each rat, three distinct slides were examined and evaluated at 10 × and 40 × magnifications.

#### Imaging analysis for assessing immunohistochemical data

Using ImageJ software, the immunohistochemical responses of caspase-3 and PCNA were identified in ten sections of ovarian tissue. The image analyzer converts the pixels into area percentages. The results are presented as the mean ± SD. A value of *P* ≤ 0.05 was considered to indicate a statistically significant difference between groups.

## Statistical analysis

One-way analysis of variance (ANOVA) was used to compare the results. After that, Tukey’s test was performed on several groups. [SPSS software (version 20)]. *P* values ≤ 0.05 were considered to indicate statistical significance. The data are presented as the mean ± SD.

## Results

### Characterization of nanoparticles

**Fig. 1:**
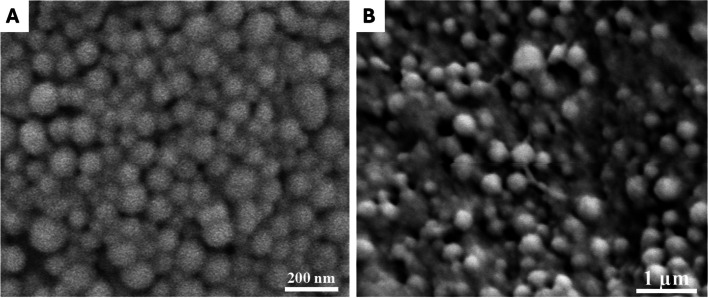
SEM images of (**A**) Ag-NPs and (**B**) CS-SeNPs. The scale bars represent 200 nm and 1 μm

**Fig. 2:**
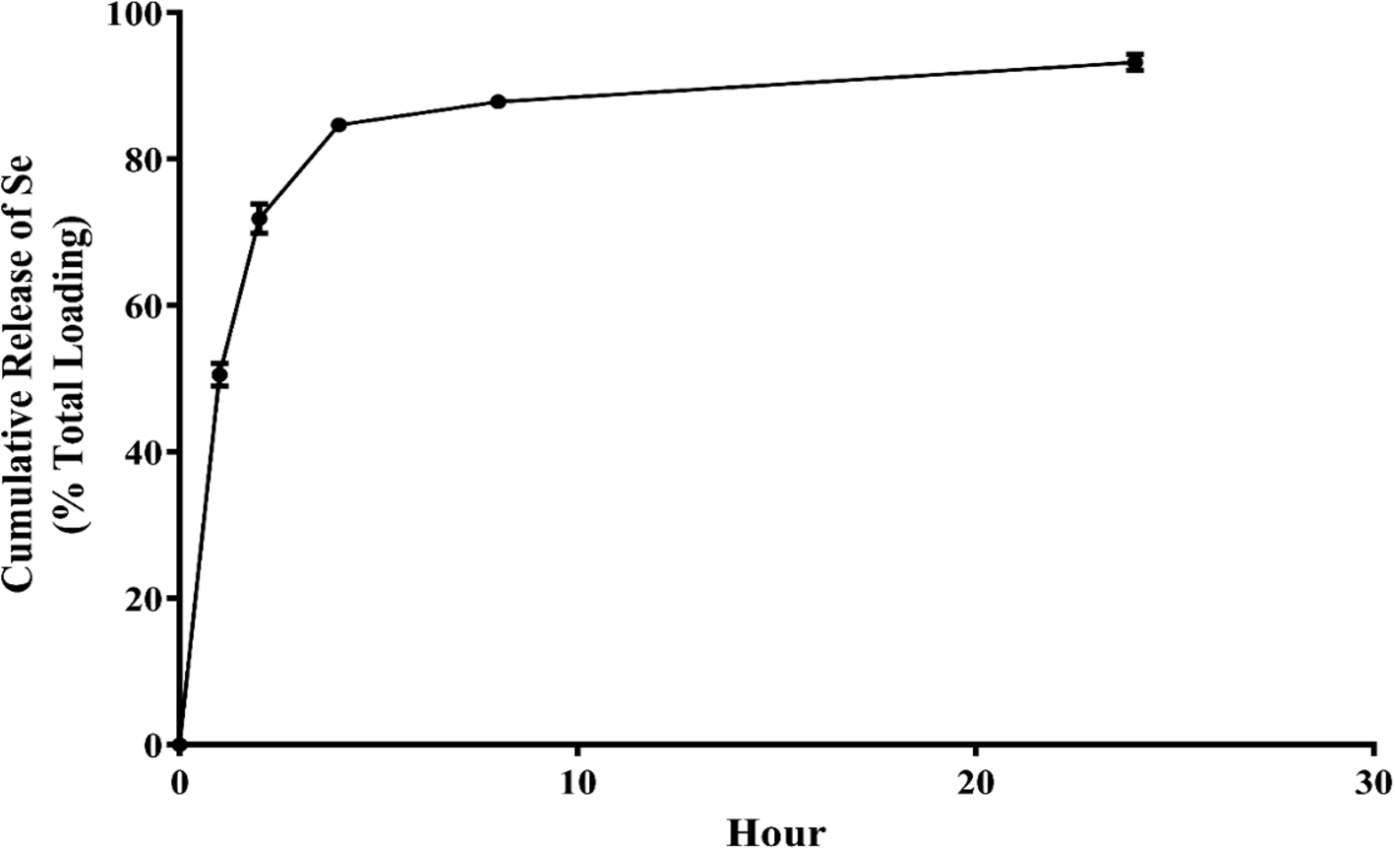
The release of selenium from chitosan nanoparticles over a day. The data are displayed as the mean ± SD (n = 3)

The morphologies of the silver nanoparticles (Ag-NPs) and selenium-loaded chitosan (CS-Se NPs) were determined via SEM images. Additionally, the average diameters of the Ag-NPs and CS-SeNPs were 44.6 ± 8.5 nm and 241.6 ± 26.8, respectively. The Ag-NPs and CS-SeNPs had zeta potentials of −13.2 ± 0.4 mV and + 42.6 ± 0.5 mV, respectively (Fig. 1). Chitosan showed high encapsulation of selenium (Se) with an encapsulation efficiency (EE) of 87.8 ± 4.4%. The selenium was released in a surge during the first four hours of incubation (84,7 ± 1.7%) and then gradually decreased until the end of the experiment at 24 h (Fig. 2).

### Biochemical investigation (oxidative stress parameter measurements)

#### Reduced glutathione (GSH)

**Fig. 3:**
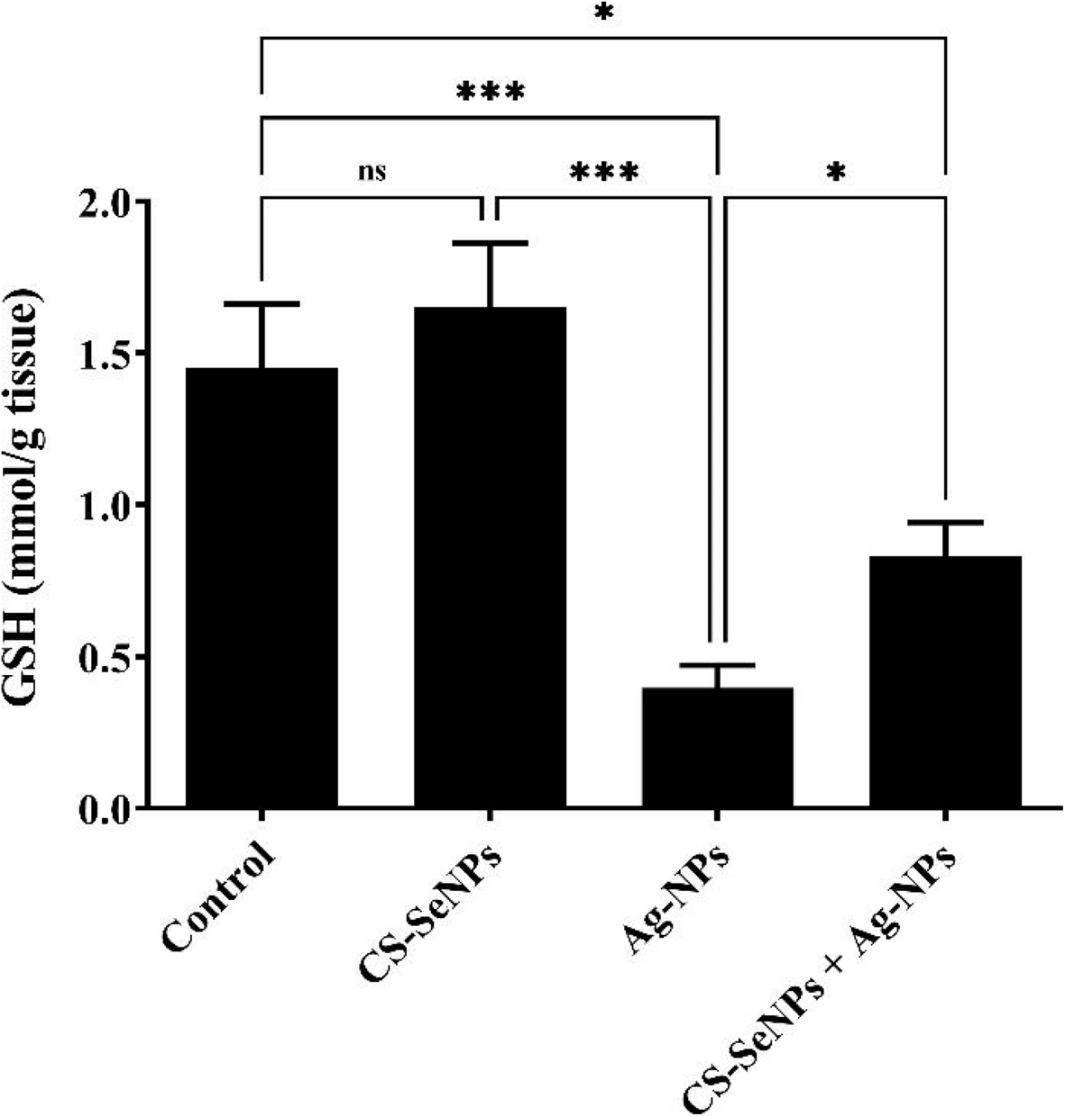
Reduced glutathione (GSH) levels in the ovarian tissue of rats were measured in three groups: those exposed to Ag-NPs, those treated with both CS-SeNPs and Ag-NPs, and those treated with CS-SeNPs alone. The data are presented as mean ± SD (n = 7). Statistical significance is indicated as nonsignificant (ns), *P <0.05, and ***0.001

Compared to those in the control group, our findings showed a substantial (*P* < 0.001) decrease in the level of ovarian GSH in the group exposed to silver nanoparticles (Ag-NPs). Conversely, compared to that in the Ag-NPs exposed group, the GSH concentration was significantly (*P* < 0.05) greater in the group that was administered both Ag-NPs and CS-Se NPs (Fig. 3).

#### Superoxide dismutase (SOD)

**Fig. 4:**
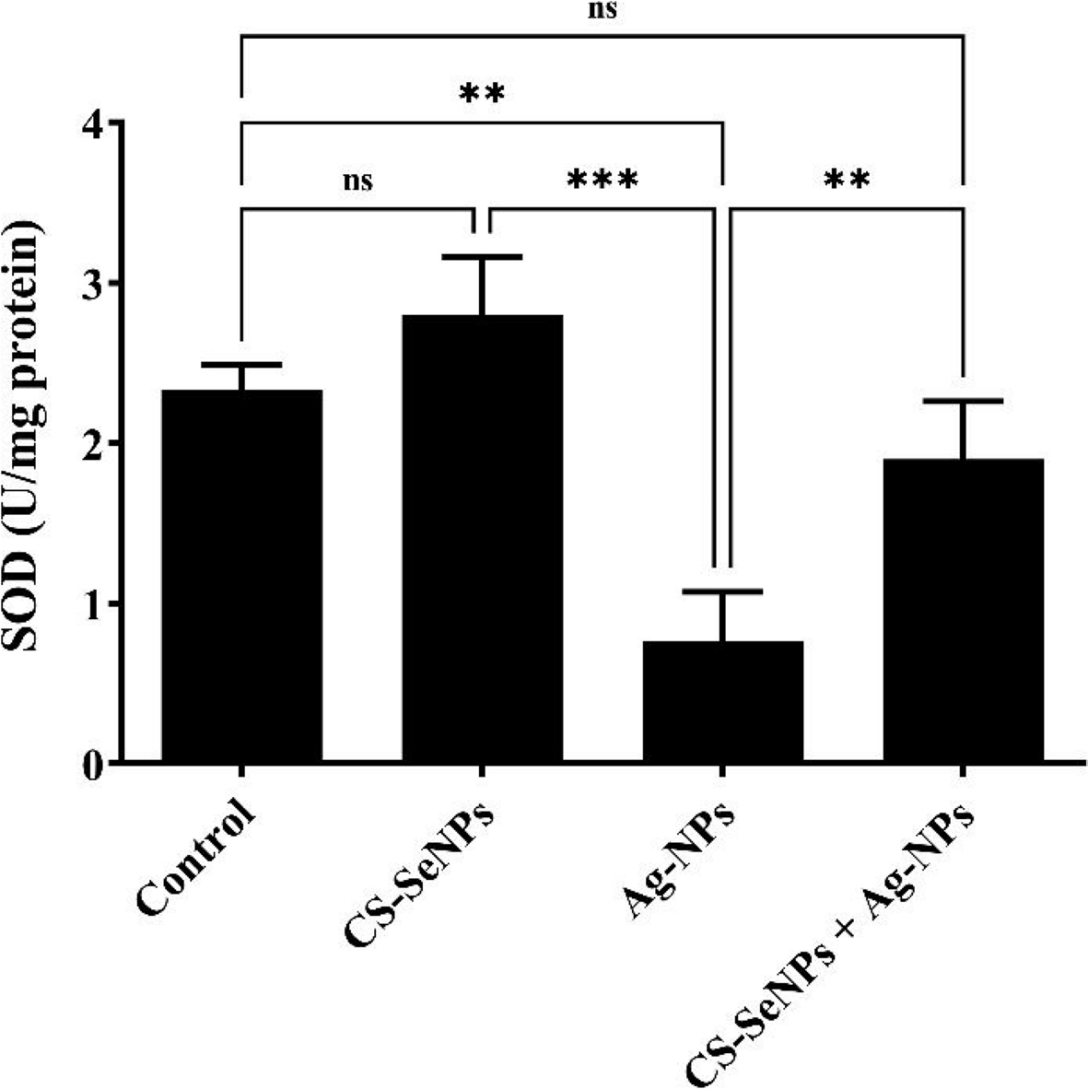
Superoxide dismutase (SOD) activity in the ovarian tissue of rats exposed to Ag-NPs, (CS-SeNPs + Ag-NPs), and CS-SeNPs. The data are shown as the mean ± SD (*n*=7). Nonsignificant (ns); (**P<0.01; *** *P*<0.001)

According to the data presented in Fig. 4, rats exposed to Ag-NPs had significantly lower levels of ovarian SOD (*P* < 0.01) than rats in the control group. The simultaneous administration of Ag-NPs with CS-SeNPs caused a significant (*P* < 0.01) increase in SOD levels compared to those in rats exposed to Ag-NPs (Fig. 4).

### Histological and histochemical results

#### Light microscopic examination (H&E)

**Fig. 5:**
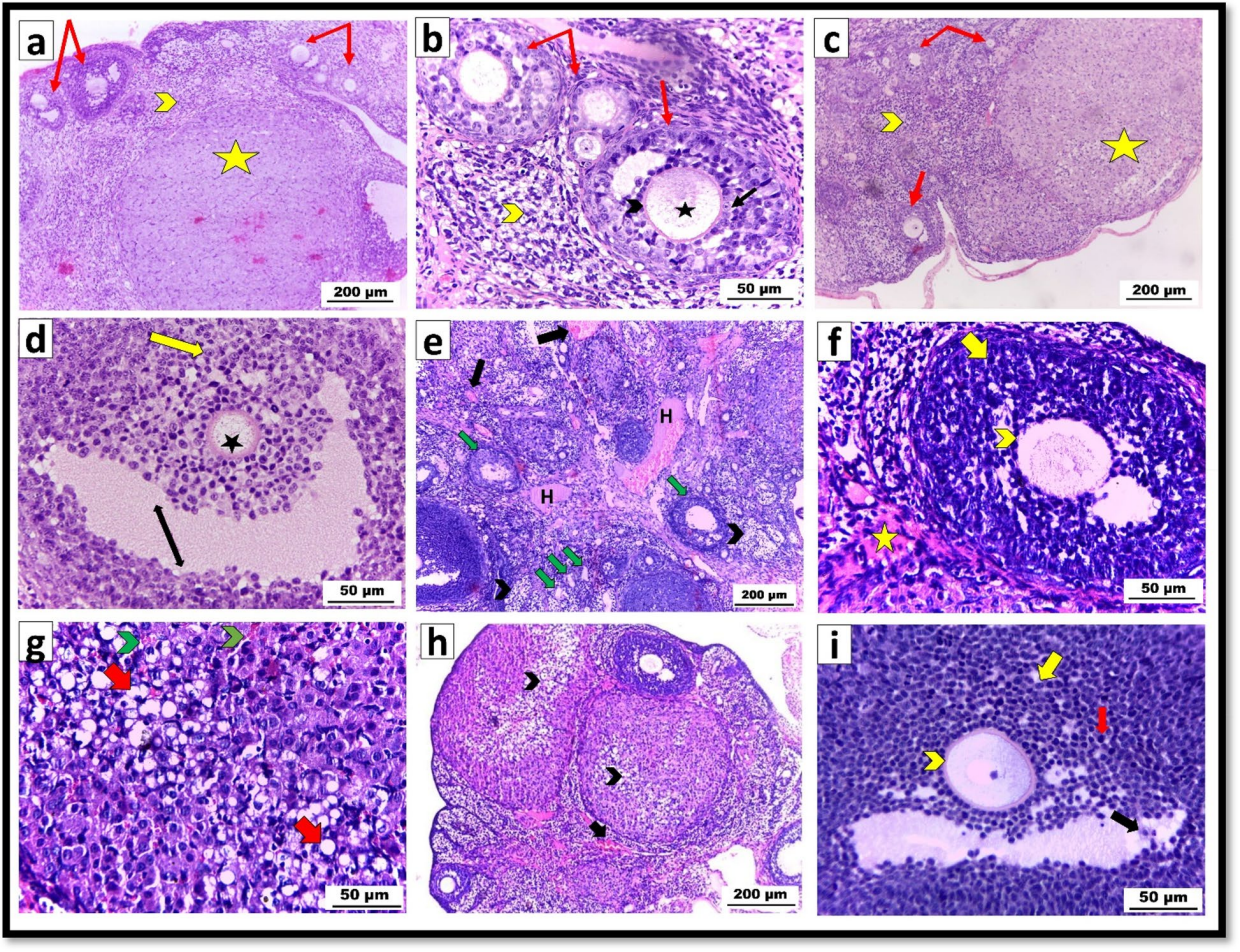
Photomicrograph of H&E-stained ovarian sections of albino rats (**a**, **b**) in the control group **a**. Normal ovarian follicles at different developmental stages (red arrows), normal stromal cells (yellow arrowhead), and a well-developed corpus luteum (yellow star) X100; **b** Normal ovarian follicles (red arrows) with normal granulosa cells having vesicular nuclei (black arrow) and a typical oocyte (black star) surrounded by a well-developed zona pellucida (black arrowhead). Normal interstitial cells were also observed (yellow arrowhead) at X400; **c**,**d** in the CS-SeNP-treated group; **c** normal ovarian follicles at different stages of development (red arrows), normal stroma (arrowhead), and a well-developed corpus luteum (yellow star) at X100 **d**. showing mature follicles with normal oocyte and typical zona pellucida (black star), normal granulosa cells with vesicular nuclei (yellow arrow), and antrum (double-headed black arrow) at X400; **e**–**g** Ag-NPs exposed group **e**. showing hemorrhage with dilated congested blood vessels (black arrows), atretic deformed follicles (green arrows), hyalinization (H), and focal areas of necrosis (black arrowheads) at X100; **f** degenerated follicle with dissociated hyperplastic granulosa cells having pyknotic nuclei (yellow arrow), degenerated oocyte with detached zona pellucida (yellow arrowhead), and fibrosis (yellow star) at X400. **g** Fatty degeneration of lutein cells (red arrow) and extravasated blood (green arrowheads) X400. **h**&**i** Ag-NPs plus CS-SeNP coadministration group **h**. recovery with small areas of fatty degeneration (black arrowhead) and a clear reduction in congestion (black arrow) X100. **i** Nearly normal ovarian follicle with a normal oocyte surrounded by an intact zona pellucida (yellow arrowhead); most of the granulosa cells exhibited vesicular nuclei (yellow arrow), while only a few cells exhibited dark nuclei (red arrow). Some detached granulosa cells were observed in the liquor folliculi (black arrow) X400

Examination of the ovarian sections stained with H&E in the control group (Fig. 5a&b) and CS-SeNPs-treated group (Fig. 5c&d) revealed the normal structure of the ovarian follicles at different developmental stages, with normal oocytes surrounded by a typical zona pellucida, normal granulosa cells with vesicular nuclei and well-developed theca interna and externa. Well-developed corpus lutea with normal stromal and interstitial cells were also observed.

In contrast, ovarian sections from Ag-NPs exposed rats revealed atretic deformed follicles, focal areas of necrosis, hyalinization, and dilated congested blood vessels (Fig. 5e). Atretic follicles appeared with dissociated and hyperplastic granulosa cells having pyknotic nuclei and degenerated oocyte with detachment of the zona pellucida. Fibrosis was also detected in the interstitial connective tissue (Fig. 5f). Excessive fatty degeneration of lutein cells and extravasated blood were also observed (Fig. 5 g).

Sections from rats coadministered Ag-NPs and CS-SeNPs showed signs of partial recovery in the form of a clear reduction in congestion. However, small areas of fatty degeneration still existed (Fig. 5 h). The ovarian follicle appeared nearly normal, with a normal oocyte surrounded by an intact zona pellucida. Most of the granulosa cells restored their normal architecture and arrangement with vesicular nuclei, whereas some granulosa cells still had darkly stained nuclei, while others sloughed into the liquor folliculi (Fig. 5i).

#### Histochemical examination (Masson’s trichrome stain)

**Fig. 6:**
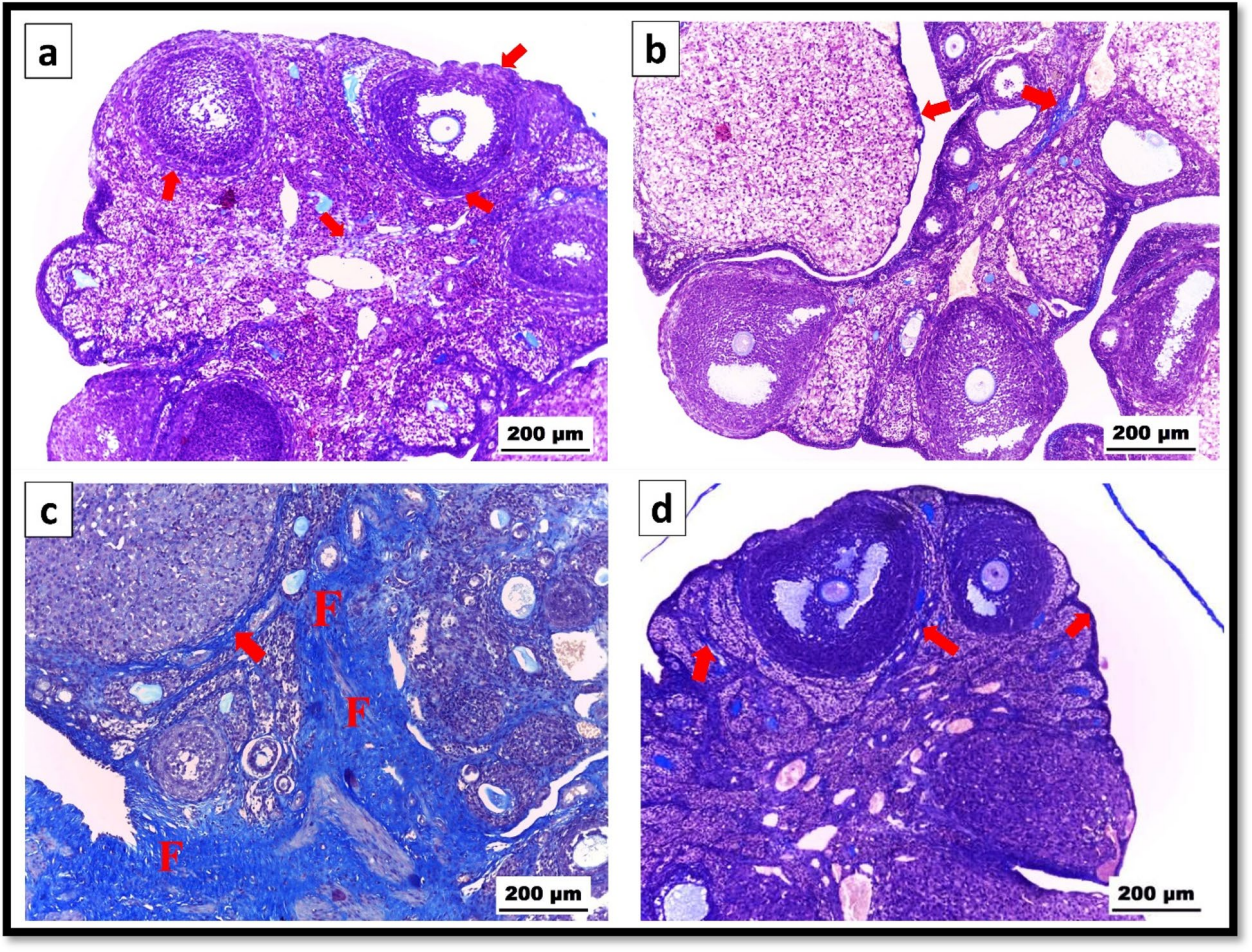
Photomicrograph of Masson’s trichrome-stained ovarian sections. **a **Control group. **b** CS-SeNPs group showing normal amounts of interstitial collagenous fibers (arrows). **c** Ag-NPs group showing extensive collagen fiber deposition (F&arrows). **d** Ag-NPs plus CS-SeNPs-cotreated group revealing moderate collagen fiber deposition (arrows) at X100

Compared to those in the control group, our findings showed that, in the capsule, corpora lutea, interstitial tissue, and surrounding ovarian follicles, almost the same quantity of collagen fibers were present in the rats that received CS-SeNPs. In contrast, the amount of collagen fibers was significantly greater when Ag-NPs were administered than in the control group, but the amount of collagen fibers was significantly lower after Ag-NPs and CS-SeNPs were coadministered (Fig. 6).

#### Quantitative investigation of the histochemical observations (Masson’s trichrome stain)

Ag-NP exposure caused a significant (*P* ≤ 0.05) increase in the amount of collagen fibers in the ovarian tissue compared to that in the control group. Additionally, when CS-SeNPs were administered alone, there was no significant change (*P* > 0.05) in the total amount of collagen fibers compared to that in the control group. Furthermore, coexposure to Ag-NPs and CS-SeNPs resulted in a significant (*P* ≤ 0.05) reduction in the number of collagen fibers compared to that in the Ag-NPs group (Table 1).

**Table 1:** Impact of Ag-NPs and/or CS-SeNPs on the area (µm2) of rat ovarian tissue specimens coated with collagen fibers

Groups	Control	CS-SeNPs	Ag-NPs	Ag-NPs + CS-SeNPs
Masson’s trichrome stain (µm^2^)	82.39 ± 5.63 ^**b, c**^	82.44 ± 8.75 ^**b, c**^	219.61 ± 13.20 ^**a, c, d**^	173.19 ± 12.52 ^**a, b, d**^

The values (*n* = 10 rats/group) are shown as the mean ± SD. A *P* value ≤ 0.05 indicates a significant difference between means in the same row with different small superscript letters

### Immunohistochemical results

#### Immunohistochemical results for caspase 3

**Fig. 7:**
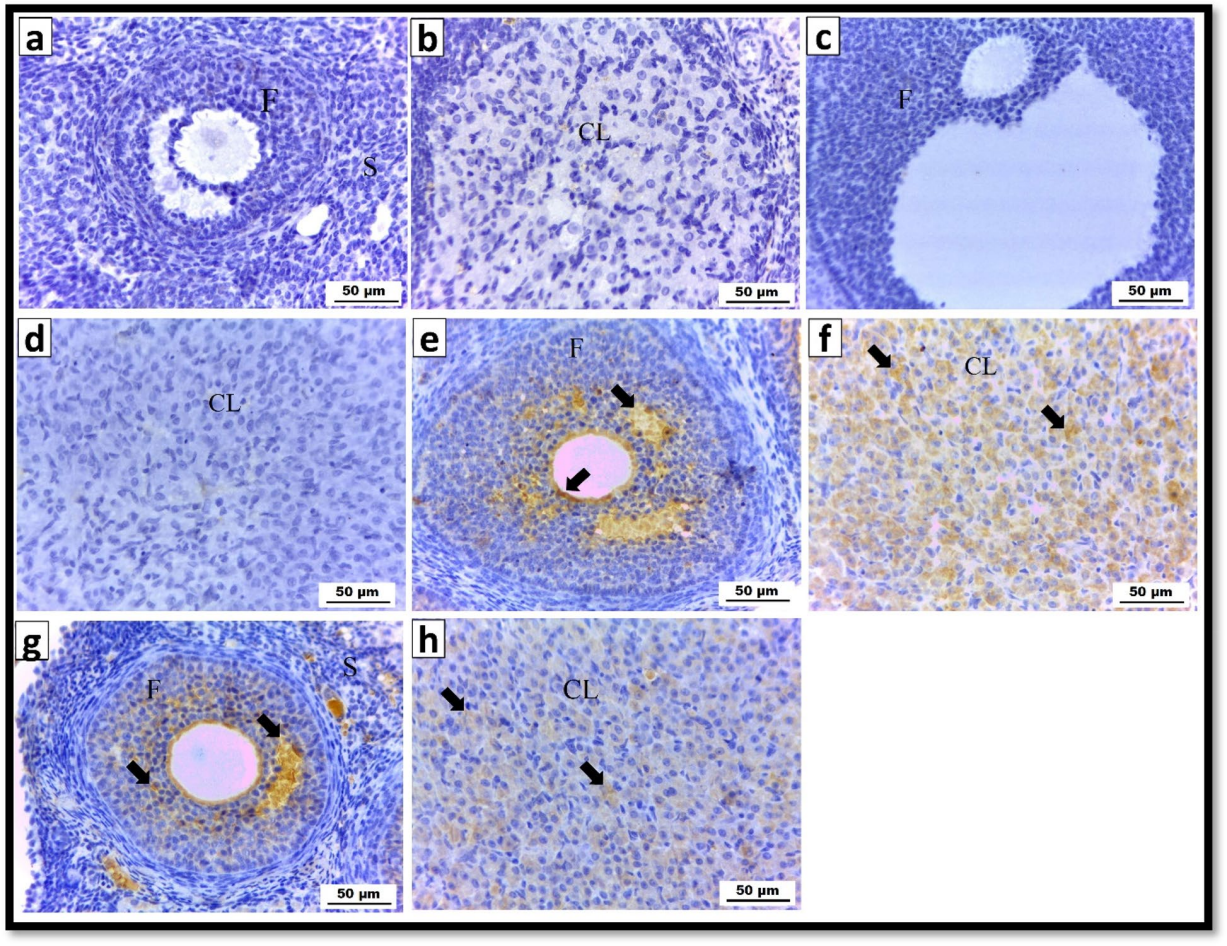
Photomicrograph demonstrating the immunohistochemical reactivity of caspase-3 in albino rat ovarian sections (X400). **a**, **b** Control group; **c**, **d** CS-SeNPs exposed group showing a mild (+) caspase-3 immune reaction. **e**, **f** Ag-NPs exposed group revealing strong positive (+++) caspase-3 immune expression (arrows). **g**, **h** The group coadministered Ag-NPs and CS-SeNPs demonstrated a moderate (++) caspase-3 immune response (arrows)

Ovarian tissue sections from the control group (Fig. 7a&b) and the group that received CS-SeNPs (Fig. 7c&d) showed mild positive ( +) immunoreactivity for caspase-3 when subjected to immunohistochemical evaluation. On the other hand, strong positive (+ + +) immunoreactivity for caspase 3 was observed in Ag-NPs exposed rats (Fig. 7e&f), while a noticeable decrease in immune expression was observed in the rats coexposed to Ag-NPs combined with CS-Se NPs (Fig. 7 g&h).

#### Immunohistochemistry for PCNA

**Fig. 8:**
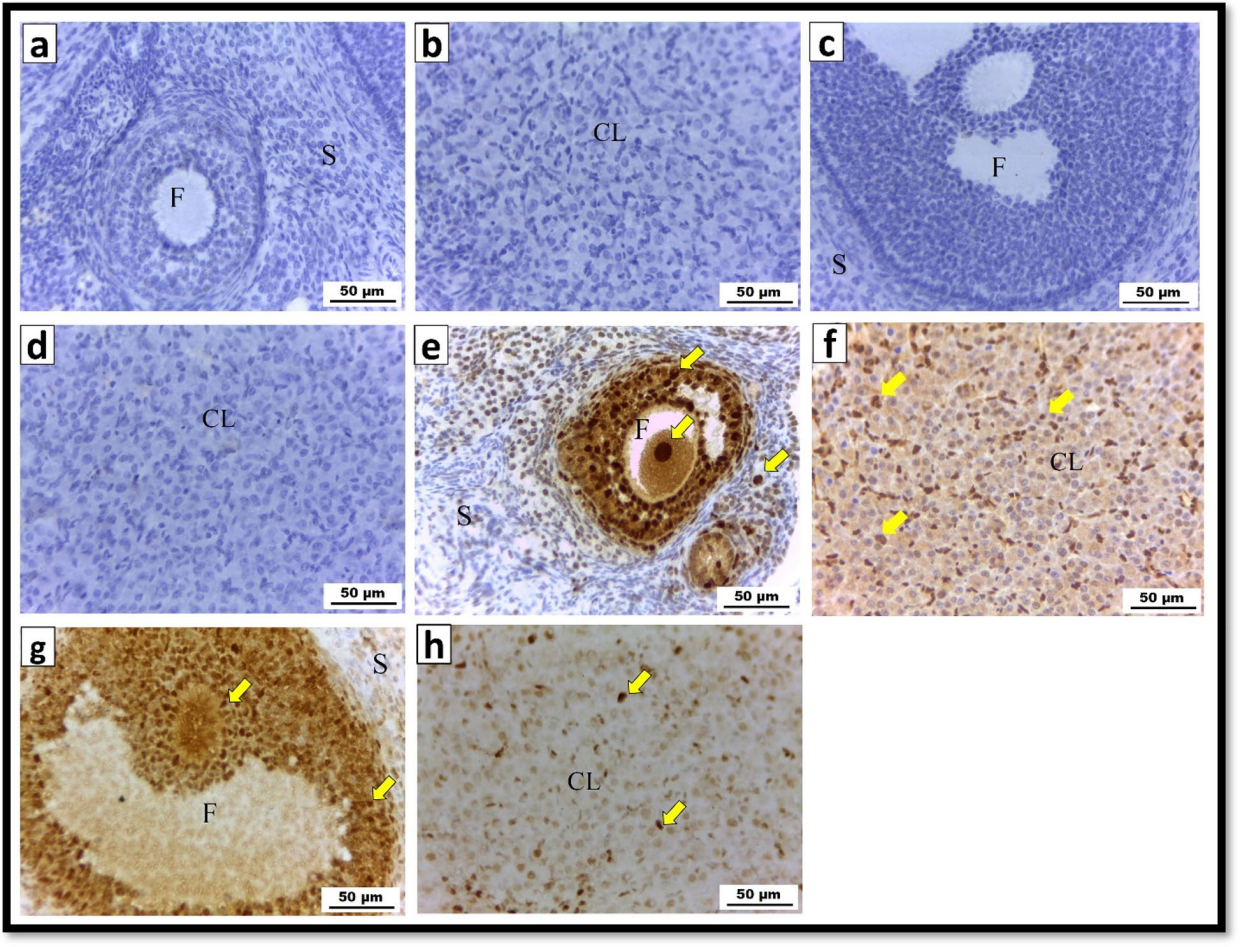
Photomicrograph of a PCNA immunohistochemical reaction in ovarian sections from albino rats (X400). **a**, **b** Control group; **c**, **d** CS-SeNPs treated group showing mild ( +) PCNA immunoreactivity (arrows). **e**, **f** Strongly positive (+ + +) PCNA immune expression was shown in the Ag-NPs exposed group (arrows). **g**, **h** The group that received both Ag-NPs and CS-SeNPs concurrently had a moderate (+ +) PCNA immunological response (arrows)

Immunohistochemical analysis of the ovarian tissue sections revealed mild (+) PCNA immune expression in the control group (Fig. 8a, b) and the CS-SeNPs exposed group (Fig. 8c, d). On the other hand, the PCNA immune reaction in the Ag-NPs exposed group was strongly increased (+++) (Fig. 8e, f). Ovarian sections from the coadministered group (Ag-NPs + CS-SeNPs) displayed moderate PCNA immunoreactivity (++) (Fig. 8g & h).

#### Quantitative analysis of the immunohistochemical studies

Comparing the ovarian tissues of the Ag-NPs group to those of the control group, there was a significant (*P* ≤ 0.05) increase in the levels of caspase-3 and the PCNA immune response. In addition, compared to that in rats treated with Ag-NPs, co-exposure to CS-SeNPs and Ag-NPs resulted in a significant (*P* ≤ 0.05) reduction in caspase-3 and PCNA immune responses. However, compared to that in the control group, the administration of CS-SeNPs only produced a nonsignificant (*P* >0.05) change in caspase-3 and PCNA immune reactivity (Table 2).

**Table 2:** The effect of Ag-NPs and/or CS-SeNPs on the area (µm2) occupied by immune-reactive cells that were caspase-3- and PCNA-positive in the rat ovarian sections

Groups	Control	CS-SeNPs	Ag-NPs	Ag-NPs + CS-SeNPs
**Caspase-3 (µm**^**2**^)	0.68±0.09^**b**^	3.02±3.93 ^**b**^	15.79±2.08 ^**a, c, d**^	4.04±1.13 ^**b**^
**PCNA (µm**^**2**^)	5.33±2.52^**b, c**^	4.67±1.53 ^**b, c**^	171.33±5.13 ^**a, c, d**^	74.00±7.94 ^**a, b, d**^

The data are displayed as the mean ± SD (*n* = 10 rats/group)

The means in the same row with different small superscript characters differ significantly at *P* ≤0.05

## Discussion

Silver nanoparticles have recently been applied to a wide range of applications. including medical device coatings, antiseptics, antibacterial agents, and antiviral agents due to their small size and potent impact [37–39]. The reproductive system is extremely sensitive to environmental hazards such as heavy metals, xenobiotics, and nanoparticles [40]. Indeed, the negative impact of Ag-NPs on folliculogenesis has been documented previously in Ag-NPs-exposed laboratory animals [15, 41].

Based on previous reports, selenium nanoparticles (Se-NPs) protect the body from metal toxicity via the effective scavenging of free radicals, as they are co-factors of antioxidant enzymes; thus, they protect cellular enzymes and nucleic acids from the damaging effects of ROS. The presence of selenium in the active sites of various antioxidant enzymes, including glutathione peroxidase, glutathione reductase, and thioredoxin reductase, is related to its essential role in the cell. Se-NPs exhibit anti-inflammatory effects by enhancing cytokine expression and reestablishing the proper ratio between the antioxidant state and oxidative stress [42–44].

Chitosan has received much attention due to its nontoxicity, biocompatibility, and biodegradability [45]. Furthermore, it can withstand pancreatic and pepsin enzymes, resulting in good bioactivity for chitosan-based nanoparticles [46].

In our study, the results of the loading of selenium onto chitosan nanoparticles revealed a small average size due to the ionic gelation technique that was applied for their manufacture [47]. Furthermore, the positive zeta potential of the CS-SeNPs preserved the stability of the nanoparticles, decreased the probability of aggregation, and encouraged electrostatic interactions with the overall negative charge of the cell membrane [48]. The residual amino groups of chitosan molecules that are entangled with the nanoparticles' surface could also cause positive zeta potential values [49]. Moreover, the chitosan nanoparticles showed significant selenium (Se) loading of 88.6 4.6%. According to earlier studies, the ratio of chitosan to tripolyphosphate (TPP) during the manufacturing of nanoparticles affects the ability of chitosan to be encapsulated; a greater TPP content during manufacture increases the effectiveness of chitosan loading [29]. The release profile of the CS-SeNPs showed that during the first four hours of incubation, selenium (Se) was released quickly, and during the next four hours, it was gradually released**.** The distribution of Se along the surface of the chitosan nanoparticles and their large surface area may account for these findings. Consequently, Se desorption from the surface of the nanoparticles could be what caused the initial burst release [50]. Following the burst release phase, as a result of polymer erosion, the release showed a gradual pattern caused by Se exposure [51].

In our study, we assessed the potential protective effect of CS-SeNPs against Ag-NPs-induced ovarian toxicity. We evaluated biochemical, histopathological, and immune-histochemical observations of immunohistochemical data from the ovarian tissue. Our biochemical investigations revealed a marked decrease in the ovarian glutathione (GSH) and superoxide dismutase (SOD) levels in Ag-NPs exposed rats, which agrees with the findings of previous studies. This could be explained by the ability of Ag-NPs to release excessive reactive oxygen species (ROS) that induce oxidative stress, which causes cellular macromolecule damage, such as DNA, lipids, proteins, and carbohydrates, and hence cell death [52, 53]. Oxidative stress occurs when ROS exceeds the capacity of cellular endogenous antioxidant enzymes (superoxide dismutase, catalase, and GSH), which defend the cell from free radical attacks [54]. The depletion of GSH in Ag-NPs exposed rats may be associated with the direct conjugation of Ag-NPs metabolites to glutathione as GSH is responsible for the detoxification of increased ROS and peroxides [55].

In contrast, the administration of CS-SeNPs in combination with Ag-NPs significantly increased the ovarian level of GSH and SOD. These results are consistent with the results of [56]**,** who reported that SeNPs significantly elevated SOD and GSH levels in hepatic and renal tissue homogenates when concurrently administered with AlCl3. Several studies have shown that Se is important for maintaining fertility. Se deficiency can result in a fragile midpiece [57] and lead to cystic ovaries [58, 59]. Furthermore, many studies have shown that Se improves animal reproductive performance by integrating selenium into the structures of selenoproteins and selenoenzymes, such as glutathione peroxidase (GPx), which can absorb ROS and prevent the onset of oxidative damage to cells [60]. The cationic properties of chitosan allow it to electrostatically interact with anionic medications to create ionic complexes [61]. Moreover, the use of chitosan nanoparticles as a coating agent can improve drug absorption and protect against gastrointestinal system deterioration [62]. Furthermore, coating nano selenium with chitosan protects cells against DNA damage caused by selenium [24].

Our histopathological observations revealed that Ag-NPs caused many pathological alterations in ovarian tissue in the form of atretic deformed follicles, which can be explained by the ROS generation activity of Ag-NPs, which caused a marked reduction in ovarian vascular distribution and downregulation of ovarian angiogenesis, inducing atresia in the follicles [53, 63]. High dissociation of granulosa cells and their pyknotic nuclei was the most common sign of ovarian structural damage, which is in agreement with the findings of [14, 64, 65]and [66]. [67] reported that Ag-NPs cause DNA breakdown and damage, resulting in pyknosis of germ cell nuclei. The observed fatty degeneration in the present study was due to the detrimental effect of Ag-NPs on granulosa luteal cells, which aligns with the findings of previous studies [68].

Administration of CS-SeNPs is a theoretically protective approach for reducing gonadotoxicity and improving histopathological alterations in the ovary induced by Ag-NPs, as evidenced by our microscopic results showing partial recovery of the ovarian parenchyma, which was probably due to the ability of CS-SeNPs to boost cellular antioxidant functions and remove the generated ROS induced by Ag-NPs toxicity.

The present study showed excess fibrosis in the ovarian tissue of the Ag-NPs exposed group, which is in agreement with the findings of [69] and [70]**,** who reported that Ag-NPs cause excessive collagenous fiber deposition in testicular and renal tissues [71]. assumed that Ag-NPs damage ovarian blood vessels, so the blood supply to some regions of the ovarian tissue is cut off, resulting in focal fibrosis with damage to the ovarian follicles. Collagen fiber deposition was markedly decreased in the group concurrently exposed to both Ag-NPs and CS-SeNPs, which is consistent with the [2] who revealed that Se-NPs markedly reduce the amount of collagen fiber deposition that is increased by chromium in the thyroid gland.

Caspase-3 belongs to the cysteine-aspartic acid protease family and is involved in apoptosis by initiating signaling cascades between death-promoting stimuli and protein substrate cleavage sites [60]. To further understand the mechanism of CS-SeNPs-induced apoptosis, we measured the expression of the apoptotic caspase-3 protein. The present immunohistochemical results revealed that Ag-NPs strongly promoted caspase-3-mediated immune reactions in ovarian tissue, which is in agreement with the findings of previous studies [72]and [65]. These findings confirmed the belief that apoptosis, which involves the loss of cell membrane potential, is related to Ag-NPs-mediated cell death [73].

Conversely, coadministration of CS-SeNPs significantly reduced the immune reaction to caspase-3 [31] reported that the administration of CS-SeNPs with Ag-NPs decreased caspase-3 in brain tissue. Additionally [74], reported that chitosan markedly reduced caspase-3 immune expression in testicular tissue when it was administered with TIO2NPs.

Proliferating cell nuclear antigen (PCNA) is a DNA clamp required for replication that serves as a processivity factor for DNA polymerase in eukaryotic cells [75]. A significant increase in PCNA immune expression was observed in the rats after Ag-NPs exposure attributed to an increase in the mitotic activity of the ovarian cells following the toxic insult induced by Ag-NPs. However, when Ag-NPs and CS-SeNPs were administered simultaneously to rats, the immunological expression of PCNA decreased, which demonstrated the potent anti-hyperproliferative effects of the CS-SeNPs. According to [76], when selenium nanoparticles (SeNPs) were administered as a preventative and therapeutic supplement, the number of PCNA-positive cells effectively decreased.

## Conclusion

Despite the excessive use of Ag-NPs in contraceptives and other gynecological products, worries about their toxicity increase, particularly in the long run. We verified that supplementation with CS-SeNPs can effectively ameliorate the harmful impact of Ag-NPs on ovarian tissue, as indicated by a clear reduction in antioxidant enzymes and many histopathological alterations with strong positive caspase-3 and PCNA immune expression. On the other hand, the coadministration of CS-SeNPs improved most of the negative effects induced by Ag-NPs by reestablishing the cellular redox state. Moreover, the activity of selenium increased in response to the chitosan coating. These findings indicate that CS-SeNPs supplementation may offer protection against the ovarian toxicity induced by Ag-NPs.

## Data Availability

No datasets were generated or analysed during the current study.

## Abbreviations

Ag-NPs: Silver nanoparticles
CS-SeNPs: Selenium-loaded chitosan nanoparticles
GSH: Reduced glutathione
GPx: Glutathione peroxidase
SOD: Superoxide dismutase
ROS: Reactive oxygen species
CS: Chitosan
Se: Selenium
SEM: Scanning electron microscopy
XO: Xanthine oxidase
WST-1: Water-soluble tetrazolium
LM: Light microscopy
H&E: Hematoxylin and eosin
PCNA: Proliferating cell nuclear antigen
Ns: Nonsignificant
